# The Treatment of Neuralgia in General Practice

**Published:** 1887-11

**Authors:** Gustavus Eliot

**Affiliations:** New Haven, Conn.


### *THE TREATMENT OF NEURALGIA IN GENERAL PRACTICE.**

By GUSTAVUS ELIOT, A. M., M. D., New Haven, Conn.

No disease of the nervous system comes under the care of the general practitioner more frequently than neuralgia. Concerning the treatment of no common disease, with the possible exception of dyspepsia, are the directions contained in the popular text-books more inharmonious and indefinite. A writer often speaks in terms of the highest praise of some drug which other authors scarcely mention; while other drugs, to the value of which many have testified, is simply named—without any remarks in regard to the special indications for its use, the proper dose, the necessary frequency of administration, and the length of time for which it should be continued.

*Read before the Section of Psychological Medicine and Diseases of the Nervous System, of the Ninth International Medical Congress, at Washington, D. C., Thursday morning, September 8, 1887.

Most of the recent contributions to the therapeutics of neuralgia—and they have not been few—have been descriptive of drugs or remedial measures which are not applicable to ordinary cases, but have proved useful in cases presenting unusual characteristics, or in cases which might have been cured with equal, and perhaps greater, promptness by the use of better-known and well-established remedies. It is not of the former—the unusual—cases, nor of peculiarly obstinate cases, that this paper is intended to treat, but of the ordinary acute attacks, which lead the sufferers to consult a physician at the outset of the disease, when a prompt, complete and inexpensive cure is demanded. Active and efficient remedies are requisite for such patients. Often they see the physician but once, and expect that a single prescription will effect a cure. What plan of treatment shall be adopted at the first consultation? What remedies are the most reliable? How far can they be depended upon? Such problems as these again and again confront the general practitioner.

Before entering the strict confines of the subject, it is desirable, in order that the grounds upon which some of the conclusions are based may be understood, that some general observations should be made concerning the nature and relations of neuralgia. As commonly seen in practice, it is, so far as has been demonstrated, a purely functional disturbance of the affected nerves. The painful phenomena due to pressure and neuritis are of a different nature, and deserve separate consideration. Almost any of the sensory nerves may be affected. Those which suffer most frequently are the different branches of the trigeminal, cervical, brachial, intercostal, lumbar and sciatic nerves. Certain recurrent paroxysmal affections of the visceral nerves are also truly neuralgic. Not every case of colic or spasmodic affection of the hollow organs and passages of the abdominal and pelvic cavities should, however, be included under this head. On the other hand, that painful, unilateral, paroxysmal affection of the head, known as migraine, may fairly be classed as neuralgic.

In order to select a rational plan of treatment, it is necessary to have some definite ideas in regard to the relation which the disorder in question bears to certain other diseases and abnormal states. One of the most important conditions which favor the development of neuralgia is an alteration of the blood, and particularly a deterioration of its quality. Romberg said: "Pain is the prayer of a nerve for healthy blood," and in a large proportion of the cases of neuralgia, the influence of anæmia is very striking. Especially in chronic and obstinate forms of the disease, is a recognition of its influence an important pre-requisite for successful treatment.

The presence of certain abnormal substances in the blood may directly, or in connection with other pre-disposing conditions, cause neuralgic manifestations. The contamination of the blood by the products of certain abnormalities of digestion and assimilation, as well as by certain excrementitious substances which have escaped elimination, deserves particular mention as an etiological factor. Certain morbific agents, which ordinarily cause other manifestations, may also occasionally give rise to a neuralgic attack.

In certain sections of this country—among which is Connecticut, the residence of the writer—it has become customary to describe as malarial nearly every disease which shows an intermittent or remittent character, and to consider this diagnosis of the etiology of the affection as completely demonstrated, if benefit follows the administration of the cinchona alkaloids. Many seem to be ignorant of, or to forget, the fact (to which Dr. David Clarke, of Toronto, called the attention of this section on Tuesday morning,) that neuralgia is a paroxysmal disease, and is characterized by remissions and intermissions. When it is remembered that quinine is one of the most active tonics and stimulants of the nervous system, it will readily be seen that the influence of quinine upon the neuralgic symptoms must be regarded as of little value in proving or disproving their malarial origin. Unquestionably, some cases are due to malarial poisoning, but the ratio which these bear to the total number is much smaller than it is generally believed to be.

Another less prevalent, but equally erroneous, view attributes a rheumatic origin to many cases of neuralgia. The truth of the matter is, that such a relationship can be definitely established only with extreme infrequency.

Syphilis—the pains following syphilitic periostitis, and the osseous pains being excluded—occasionally, though not very commonly, can be recognized as causing true neuralgia.

A lithæmic condition somewhat pre-disposes to painful affections, including neuralgia, generally of a sub-acute or chronic character.

Equally important with these anæmic and toxæmic influences, and frequently associated with them, stands the influence of an excessive expenditure of nervous energy. Fatigue or exhaustion, either physical or mental, strongly pre-dispose to attacks of neuralgia. In much the same way, cold and dampness call for an extra amount of nervous energy, in order to maintain the usual functional power of the nerve, and when this is not forthcoming, neuralgia often follows.

The dependence of neuralgia upon reflex causes is a subject of considerable complexity and uncertainty. The most common and obvious illustration of this relationship is the dependence of trigeminal neuralgia upon caries of the teeth. This is often proven conclusively by the relief which follows the extraction of the diseased tooth.

Of all the drugs which have been recommended and tried in the treatment of neuralgia, three are invaluable. They are morphine, quinine and iron. In acute attacks of marked severity, morphine is almost indispensable. It is preferably administered, if the patient is seen while severe pain continues, by hypodermatic injection. Given by this method, in doses of one-quarter or one-half grain, at the onset, or during the persistency of a paroxysm, the relief which follows is prompt, grateful, and often complete. Thus used, it is always palliative, and sometimes curative, a second attack never occurring.

It is, however, rather unusual to see the course of neuralgia immediately arrested by this treatment, if it is commenced after more than one paroxysm has occurred. In these cases, it is desirable to prescribe, if possible, some remedy which is more directly curative. The drug which acts more positively in this direction than any other, which more justly than any other might be called a specific, is quinine. It should be given in large doses, and is of value in all cases, but especially in those which are markedly paroxysmal, and most of all in those which are dependent upon malarial poisoning. The relation of neuralgia to this cause, and the necessity of giving large doses of quinine in order to effect a cure, are illustrated by the history of one of the first cases which came under my observation six years ago, during my service as resident physician at the New York City work-house.

*Case of Malarial Supraorbital Neuralgia.*—J. C., male, thirty-two years old, while under treatment for an ulcer of the leg suffered an attack of intermittent fever, which was promptly cured. A few days later, he had an attack of neuralgia, affecting the right supraorbital nerve, which recurred every morning. Fifteen-grain doses of sulphate of cinchonidia, given early each morning, had no effect upon the recurrence or severity of the paroxysms. The dose being doubled, and thirty grains given at once, no further paroxysms occurred. He remained in the hospital two months, during which he enjoyed immunity from the disease.

Similar observations as to the efficacy of large doses of quinine have been recorded by different authors with regard to cases in which a malarial origin could not be detected.

In a considerable proportion of cases, anæmia is not a prominent feature. The patients are well-nourished and full-blooded subjects. In them, neuralgia is an expression of an exhaustion of nervous force, perhaps due to cold or to over-exertion; or of a toxæmia, perhaps malarial; or of some reflex irritation. In the latter cases, the removal of the source of irritation is, of course, indicated. In the other cases, in connection with proper hygiene, including, as of highest importance, rest and good food, a combination of quinine and morphine is of the greatest value.

In such cases, seeing the patient in the interval between the paroxysms—when, of course, the hypodermatic use of morphine is not indicated—it is often convenient to give the two drugs together. One drachm of sulphate of quinine and one grain of sulphate of morphine, having been thoroughly mixed, may be divided into twelve powders. Of these, one may be given two or three hours after each meal, and two (or more, if necessary,) at once, one or two hours before the paroxysm is expected.

In another large class of cases, anæmia is the predominant characteristic. These cases are often exceedingly obstinate, and likely to recur. For them, no drug is more valuable than iron. When given continuously, as the quality of the blood improves, the neuralgic tendency grows weak.

As remedies of secondary value, may be classed gelsemium and aconite. These drugs have both been very highly commended for the cure of neuralgia. They certainly deserve to be ranked as valuable adjuncts to the remedies previously named. The danger of producing unpleasant toxic effects with either drug is, however, so great as to render it inexpedient to rely upon either as a single remedy in ordinary cases. The uncertainty in regard to the strength of the various preparations, as manufactured by different pharmacists, is also so great as to constitute a serious hindrance to the general use of either drug. Furthermore, the varying susceptibility of different patients introduces another element of uncertainty. I desire, however, to bear personal testimony to the utility of five-drop doses of the fluid extract of gelsemium, repeated every four hours, and given in connection with quinine and morphine. These three together will often give most favorable results.

As remedies of the third class, may be mentioned arsenic, nux vomica, belladonna, phosphorus and iodide of potassium. These have long been highly commended. I name them rather in deference to popular opinion than because I consider them of great value. I have used them all, but have failed to obtain with them results which warrant me in commending them. Arsenic, in particular, has been recommended by many authors. I have never seen very marked improvement follow its use. On the contrary, I have seen symptoms of considerable severity develop in spite of its administration in full doses, advised with the hope of preventing the attack. As an adjuvant of iron in cases of anæmia, it is unquestionably of value in curing the anæmia. Thus, indirectly, it may contribute to the cure of neuralgia. Similarly, nux vomica and its alkaloid, strychnine, are of use as stomachic tonics, when the general nutrition is impaired by indigestion.

External applications are of considerable utility as adjuvants to internal medication. Counter-irritants have been largely used. In obstinate cases, particularly of sciatica, blisters and cauterization sometimes do great good. In cases of moderate severity —as in intercostal neuralgia—sinapisms, applied either over the seat of pain, or over the origin of the affected nerve, often afford some relief. Various sedative applications have some value as palliatives. I have frequently used a mixture containing one part of oil of gaultheria and two parts of olive oil, or a combination of equal parts of oil of origanum, tincture of opium, spirit of ammonia and olive oil—known as Fahnestock's liniment—warmed and thoroughly rubbed along the course of the affected nerve and its branches, hot flannel being superposed, and have found that either will produce a palliative effect in many instances.

Electricity is of unquestionable value in the treatment of neuralgia. The majority of cases, however, get well without it. In order that it may be used satisfactorily, it is necessary that the practitioner should own expensive and cumbersome apparatus, and that he should have sufficient special knowledge to enable him to keep it in order, and to use it. As the majority of neuralgic cases cannot be induced to make frequent and regular visits to the physician's office, and as many of them are seen only at their homes, often at long distances and at not very brief intervals, it is apparent that electricity can hardly be classed among those remedies of which the general practitioner would be likely to make early trial.

In the treatment of sub-acute and chronic cases, occurring in over-worked, under-fed and half-rested subjects, in whom indigestion is a frequent accompaniment of the neuralgia, remedies applicable to the digestive disturbances are to be used, with general tonics, in addition to the drugs which act more directly upon the nervous system. Useful combinations of this kind include aloes, strychnine, arsenic, quinine, iron and phosphide of zinc, or gelsemium, with cardamon, nux vomica and cascara sagrada.

In conclusion, I wish to emphasize the impracticability of relying upon any single drug or therapeutic measure in the treatment of neuralgia. The most useful drugs produce the best results only when given in such large doses that there is danger of the production of unpleasant symptoms. It is, therefore, far more wise to combine different remedies in such a way as to effect a cure without causing the patient discomfort. Morphine, quinine and either gelsemium or iron, with warm external applications, will prove useful in the majority of acute cases. In sub-acute and chronic cases, the morphine should be omitted, and stomachic and intestinal tonics substituted therefor.

